# Subjective task load and psychological ownership in generative AI collaborative music creation: mechanisms shaping creators’ state sense of agency

**DOI:** 10.3389/fpsyg.2026.1835406

**Published:** 2026-05-01

**Authors:** Wenting He

**Affiliations:** Zhengzhou University, Zhengzhou, China

**Keywords:** AI automation level, generative AI, human-AI co-creation, musical expertise, psychological ownership, state sense of agency, subjective task load

## Abstract

Generative artificial intelligence (AI) has transitioned into a collaborative creator in music composition, raising concerns about its impact on human creative agency. This study investigates how AI automation levels affect creators’ state sense of agency, examining the serial mediating roles of subjective task load and psychological ownership, and the moderating role of musical expertise. A 3 (AI automation level: low, medium, high) × 2 (musical expertise: novice, expert) between-subjects experiment was conducted (*N* = 162). Participants completed a music co-creation task using standardized AI generative tools. Results revealed that higher AI automation significantly reduced subjective task load, psychological ownership, and state sense of agency. Furthermore, subjective task load and psychological ownership serially mediated the negative relationship between AI automation and state sense of agency. Musical expertise significantly moderated these effects, with experts experiencing a more pronounced decline in psychological ownership and agency under high automation compared to novices. These findings indicate that while high automation reduces creative effort, it alienates creators from their output by diminishing process involvement and belongingness. Future generative AI systems should balance efficiency with agency preservation to support sustainable human-AI co-creation.

## 1 Introduction

Over the past 5 years, generative artificial intelligence has transitioned from an auxiliary tool to a collaborative creator in music composition. New systems provide comprehensive structural suggestions via multimodal inputs. This expands creative access and alters how users organize inspiration and develop concepts (Choi et al., 2025; Kim et al., 2025). Generative AI redistributes core human tasks like ideation, exploration, and integration. Studies indicate that individuals show better creative performance and subjective experiences when acting as co-creators rather than editors (McGuire et al., 2024). Therefore, research now focuses on the psychological mechanisms through which automation shapes human creative status.

In this context, state sense of agency (SoA) emerges as a critical psychological variable. State SoA represents the immediate experience of controlling personal actions and their outcomes (Legaspi et al., 2024). However, high automation does not guarantee stronger SoA. Process autonomy and output ownership act as essential dimensions supporting SoA (Rafner et al., 2025). Individuals easily shift from active creators to passive result selectors when algorithms preemptively assume generative control. This shift triggers significant alienation.

Current literature lacks mechanistic evidence on how automation levels shape SoA. This study systematically examines two distinct yet correlated mediators in generative music co-creation: subjective task load and psychological ownership. We empirically verify a path where AI automation levels weaken state SoA through the serial mediation of subjective task load and psychological ownership. We also compare how this mechanism differs across creators with varying expertise levels. Recent work in co-creative music AI further suggests that ownership is not merely a downstream feeling, but a design-sensitive property of the creative process. Krol et al. (2025a) showed that practising musicians’ needs in co-creative AI extend beyond output quality to broader concerns about how such systems fit real creative workflows. Relatedly, Krol et al. (2025b) found that a deep-learning-based music variation tool could support creative ownership when it relied on the musician’s own initial material and interpretive skill, thereby preserving ownership of both process and artefact. More broadly, earlier HCI work has framed AI co-creation as a user-experience problem rather than only an output-efficiency problem (Oh et al., 2018). Together, these studies suggest that the psychological consequences of automation depend not simply on whether AI assists, but on how much meaningful room remains for human initiation, elaboration, and control.

## 2 Theoretical background and hypotheses

### 2.1 AI automation level and state sense of agency

In a single music co-creation session, state sense of agency relates directly to the creators’ attribution of responsibility and satisfaction (Legaspi et al., 2024). This experience depends on a clear sense of causal dominance over melodic progression and structural arrangement. Under low automation, humans maintain control through frequent operations. However, as the AI automation level increases and the system directly outputs highly complete content, the creators’ intervention in core decisions decreases significantly. Recent studies show that agency fluctuates negatively as system takeover increases. Individuals placed in an editor role experience significantly impaired subjective control (McGuire et al., 2024; Rafner et al., 2025). H1: State sense of agency differs significantly across AI automation levels and decreases as the automation level increases.

### 2.2 Mediating role of psychological ownership

Psychological ownership is a core construct for understanding agency attenuation caused by high automation. When judging ownership of co-created outcomes, individuals rely heavily on their substantial contributions and decision-making control during the process (Xu et al., 2024). During music construction, low automation requires continuous participation, making the work an extension of personal intention. Conversely, high-automation systems preemptively take over most generative work. This compresses the creators’ process involvement, and the work is experienced more as an independent system output. Once psychological belongingness is impaired, individuals cannot attribute the generated results to their own actions, and state sense of agency inevitably declines. H2: Psychological ownership mediates the relationship between AI automation level and state sense of agency.

### 2.3 Serial mediation of subjective task load and psychological ownership

The subjective task load perceived by creators constitutes another key entry point for understanding the belongingness mechanism. Humans tend to use invested mental effort as an important cue to evaluate output ownership. This logic is also consistent with the IKEA effect, whereby labor can increase valuation and attachment to self-made products, especially when effort culminates in successful completion (Norton et al., 2012). When high-automation systems output complete results with one click, creators’ mental depletion drops drastically. This excessively low subjective task load may shift individuals from active constructors to passive responders (McGuire et al., 2024; Xu et al., 2024). A lack of sufficient participatory effort may hinder the establishment of the belief that this is my work. Therefore, automation not only intervenes directly in ownership but may also indirectly block the generation of psychological ownership by reducing subjective task load, ultimately leading to a loss of agency. H3: Subjective task load and psychological ownership form a serial mediation in the process where AI automation level affects state sense of agency.

### 2.4 Moderating role of musical expertise level

**Figure 1:**
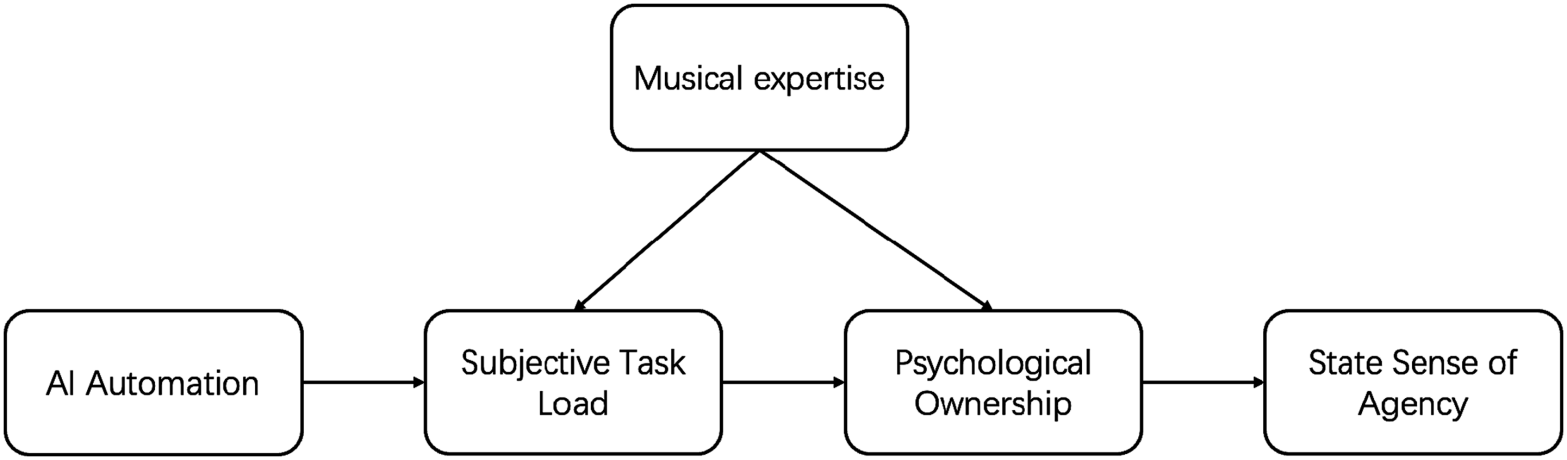
Conceptual model of the proposed moderated serial mediation.

Prior expertise level substantially alters the psychological response path to automation (Choi et al., 2025; Kim et al., 2025). Based on deskilling theory, automation empowers novices by filling technical barriers (Crowston and Bolici, 2024). However, for expert groups, high-level system takeover of core ideation directly triggers a sense of threat that their existing professional abilities are left idle. This professional identity crisis leads experts to face a more severe discontinuity in mental investment and deprivation of ownership under high automation conditions. H4: Musical expertise level moderates the effect of AI automation level on subjective task load and psychological ownership. Compared to novices, experts experience a more significant decline under high automation conditions. The proposed moderated serial mediation model is presented in Figure 1.

## 3 Methods

### 3.1 Participants

We recruited 180 university students in Henan Province, yielding a final sample of 162 (94 females, 68 males; aged 18–24, M = 20.31, SD = 1.42) after excluding 18 participants due to failed manipulation checks, missing data, technical errors, or falling within the boundary range of 3–5 years of formal music training. This final sample exceeded the *a priori* G*Power estimate of 158 for a 3 × 2 between-subjects design (*f* = 0.25, *α* = 0.05, 1 − *β* = 0.80), ensuring 27 participants per condition. Following the “six-year rule” (Zhang et al., 2020), participants were categorized solely based on years of formal music training into an expert group (*n* = 81; ≥6 years of formal training) and a novice group (*n* = 81; ≤2 years of formal training). Individuals with 3–5 years of formal training were excluded. The study was approved by the Zhengzhou University Ethics Committee. All participants provided written informed consent and received course credit.

### 3.2 Experimental design and control strategy

This study employs a 3 (AI automation level: low, medium, high) × 2 (musical expertise level: novice, expert) between-subjects design. To eliminate platform effects, all participants use Ableton Live 12 Suite with fixed native instrument presets. The AI generation environment uses Magenta Studio. This plugin includes five MIDI tools: Generate, Continue, Interpolate, Groove, and Drumify. We standardize operational permissions across groups using Max for Live templates.

The three automation levels differ only in AI generative permissions. In the low automation condition, the AI provides chord suggestions or local continuations. Participants manually develop melodies and structures. In the medium automation condition, the AI generates local motifs, rhythms, or phrases. Participants extensively modify and integrate these elements. In the high automation condition, the AI generates a complete 30-s MIDI segment based on prompts. Participants were allowed, but not required, to make one minimal edit in total, selected from a predefined set of options: replacing one motif, deleting one segment, or adjusting one instrument. This rule preserved minimal intervention rights while preventing extensive reconstruction of the AI-generated output. Interface layout, feedback speed, latency, listening procedures, and output sounds remain identical across groups. This ensures differences stem solely from the AI automation level.

### 3.3 Experimental task and procedure

All participants create a 30-s music segment under uniform conditions. We set the theme as Cyberpunk to ensure stylistic consistency. Before the experiment, researchers explain the objectives and basic operations. Participants complete a brief practice session to familiarize themselves with the interface. Subsequently, participants enter the formal creation phase. They have 20 min to compose the music segment based on the theme. After composing, participants export and listen to their final work. Finally, participants complete manipulation checks and questionnaires. We conduct the entire experiment in a unified environment to minimize confounding variables.

### 3.4 Measurement tools

Subjective Task Load. Subjective task load was contextualized for the specific task based on the Mental Demand and Effort dimensions of the NASA-TLX (Hart, 2006; Hart and Staveland, 1988). The scale consists of six items, rated on a 7-point Likert scale (1 = strongly disagree, 7 = strongly agree). Higher scores indicate a higher level of subjective task load. In this study, the Cronbach’s *α* for this scale was 0.89. The full adapted items are provided in Appendix A in Supplementary material.

Psychological Ownership. Psychological ownership was adapted into a state-based version tailored to the task context of this study, drawing upon the theory of Pierce et al. (2001) and the classic measurement approach of Van Dyne and Pierce (2004). The scale comprises six items, evaluated on a 7-point Likert scale. Higher scores indicate a stronger level of psychological ownership over the created work. In this study, the Cronbach’s α for this scale was 0.91. The full adapted items are provided in Appendix A in Supplementary material.

State Sense of Agency. State sense of agency was contextually adapted for the music co-creation task based on the state-based measurement framework of the SOARS (Polito et al., 2013). The scale includes eight items, rated on a 7-point Likert scale, with two items being reverse-scored. Higher scores indicate a stronger level of state sense of agency. In this study, the Cronbach’s α for this scale was 0.88. The full adapted items are provided in Appendix A in Supplementary material.

### 3.5 Manipulation checks and control variables

We include manipulation checks and control variables to improve internal validity. After the creation task, we measure perceived automation and perceived control. These checks verify the effectiveness of the AI automation level manipulation (Legaspi et al., 2024; Rafner et al., 2025; Xu et al., 2024).

Furthermore, we incorporated several control variables to reduce extraneous interference. DAW familiarity, prior experience with AI music tools, and familiarity with the target style were each measured using a single 7-point self-report item (1 = very low, 7 = very high). Specifically, participants rated how familiar they were with digital audio workstation software such as Ableton Live, how much prior experience they had with AI-based music generation tools, and how familiar they were with the cyberpunk style used in the task. General technology acceptance was assessed using a simplified Technology Acceptance Model approach with two 7-point items capturing perceived usefulness and perceived ease of use of AI-assisted creative systems; the mean of these items was used in the analysis. We evaluate general technology acceptance using a simplified Technology Acceptance Model approach (Davis, 1989). These controls help confirm that group differences originate from the experimental manipulation rather than preexisting experiences.

## 4 Results

### 4.1 Preprocessing, descriptive statistics, and inter-group difference analysis

Harman’s single factor test for common method bias showed that the first unrotated factor explained 28.64% of the variance. This value falls below the 40% empirical threshold, indicating no significant common method bias.

Table 1 presents the overall means, standard deviations, and correlation analysis results for all variables. AI automation level correlated negatively and significantly with subjective task load, psychological ownership, and state sense of agency. Subjective task load correlated positively and significantly with psychological ownership. Both variables correlated positively and significantly with state sense of agency. The correlation directions aligned with theoretical expectations, providing preliminary support for hypothesis testing.

**Table 1:** Means, standard deviations, and correlations among the main variables.

Variable	M	SD	1	2	3	4	5
1. AI autonomy level	2.00	0.82	—				
2. Musical expertise level	0.50	0.50	0.00	—			
3. Subjective task load	4.27	0.98	−0.42***	0.07	—		
4. Psychological ownership	4.55	0.96	−0.46***	0.09	0.41***	—	
5. State sense of agency	4.44	1.02	−0.49***	0.05	0.34***	0.60***	—

AI autonomy level was coded as 1 = low, 2 = medium, and 3 = high. Musical expertise level was coded as 0 = novice and 1 = expert. ***p < 0.001.

Table 2 shows the means and standard deviations of core variables across experimental groups. As the AI automation level increased, subjective task load, psychological ownership, and state sense of agency decreased. The expert group showed a more pronounced decline under high automation conditions than the novice group. This suggests that musical expertise level may moderate the relationship between AI automation level and creators’ psychological experiences.

**Table 2:** Descriptive statistics for the core variables across experimental groups.

Group	*n*	Subjective task load M ± SD	Psychological ownership M ± SD	State sense of agency M ± SD
Novice–low autonomy	27	4.70 ± 0.82	4.92 ± 0.79	4.96 ± 0.85
Novice–medium autonomy	27	4.31 ± 0.88	4.57 ± 0.84	4.48 ± 0.91
Novice–high autonomy	27	3.98 ± 0.93	4.03 ± 0.88	3.92 ± 0.95
Expert–low autonomy	27	4.96 ± 0.76	5.23 ± 0.72	5.18 ± 0.80
Expert–medium autonomy	27	4.38 ± 0.83	4.71 ± 0.79	4.63 ± 0.86
Expert–high autonomy	27	3.28 ± 0.97	3.82 ± 0.91	3.45 ± 1.01

To verify whether the experimental manipulation was effective, we first examined group differences in perceived automation and perceived control across the three AI autonomy conditions. The results showed a significant difference in perceived automation among the groups, *F*(2, 159) = 82.46, *p* < 0.001, η^2^ = 0.51. *Post hoc* comparisons indicated that the high-autonomy group reported significantly higher perceived automation than both the medium- and low-autonomy groups, and the medium-autonomy group also scored significantly higher than the low-autonomy group. Group differences in perceived control were also significant, *F*(2, 159) = 47.82, *p* < 0.001, η^2^ = 0.38. Post hoc comparisons showed that the low-autonomy group reported significantly higher perceived control than both the medium- and high-autonomy groups, and the medium-autonomy group also scored significantly higher than the high-autonomy group. Taken together, these results indicate that the manipulation of AI autonomy level was successful.

We then conducted a 3 (AI autonomy level: low, medium, high) × 2 (musical expertise level: novice, expert) two-way ANOVA to examine group differences in subjective task load, psychological ownership, and state sense of agency. For subjective task load, the main effect of AI autonomy level was significant, *F*(2, 156) = 18.74, *p* < 0.001, ηp^2^ = 0.19, whereas the main effect of musical expertise was not significant, *F*(1, 156) = 0.87, *p* = 0.353, ηp^2^ = 0.01. However, the interaction between AI autonomy level and musical expertise was significant, *F*(2, 156) = 4.62, *p* = 0.011, ηp^2^ = 0.06. *Post hoc* comparisons showed that the low-autonomy group scored significantly higher than both the medium- and high-autonomy groups, and the medium-autonomy group also scored significantly higher than the high-autonomy group. Simple-effects analyses further indicated that the decline in subjective task load as AI autonomy increased was more pronounced among experts.

For psychological ownership, the main effect of AI autonomy level was significant, *F*(2, 156) = 23.91, *p* < 0.001, ηp^2^ = 0.23, whereas the main effect of musical expertise was not significant, *F*(1, 156) = 0.96, *p* = 0.328, ηp^2^ = 0.01. The interaction effect was significant, *F*(2, 156) = 4.08, *p* = 0.019, ηp^2^ = 0.05. Post hoc comparisons indicated that the low-autonomy group reported significantly higher psychological ownership than both the medium- and high-autonomy groups, and the medium-autonomy group also reported significantly higher psychological ownership than the high-autonomy group. Simple-effects analyses showed that the decrease in psychological ownership under the high-autonomy condition was substantially greater for experts than for novices.

For state sense of agency, the main effect of AI autonomy level was also significant, *F*(2, 156) = 31.84, *p* < 0.001, ηp^2^ = 0.29, whereas the main effect of musical expertise was not significant, *F*(1, 156) = 0.42, *p* = 0.518, ηp^2^ = 0.00. The interaction effect was significant, *F*(2, 156) = 5.21, *p* = 0.006, ηp^2^ = 0.06. Post hoc comparisons showed that the low-autonomy group reported significantly higher state sense of agency than both the medium- and high-autonomy groups, and the medium-autonomy group also reported significantly higher state sense of agency than the high-autonomy group. Simple-effects analyses further indicated that the decline in state sense of agency under the high-autonomy condition was more pronounced among experts.

**Figure 2:**
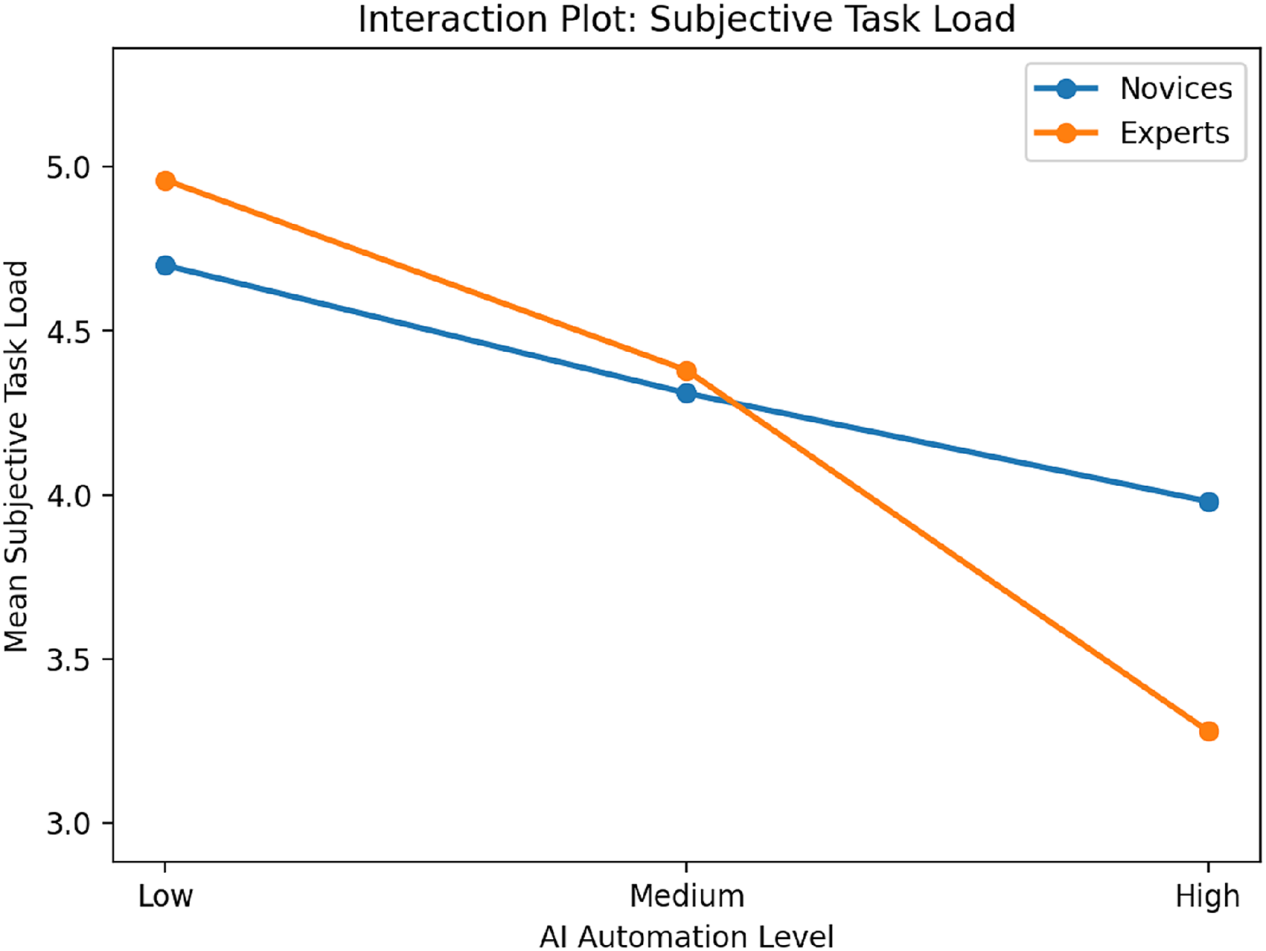
Interaction plot for subjective task load across AI automation levels and musical expertise groups.

**Figure 3:**
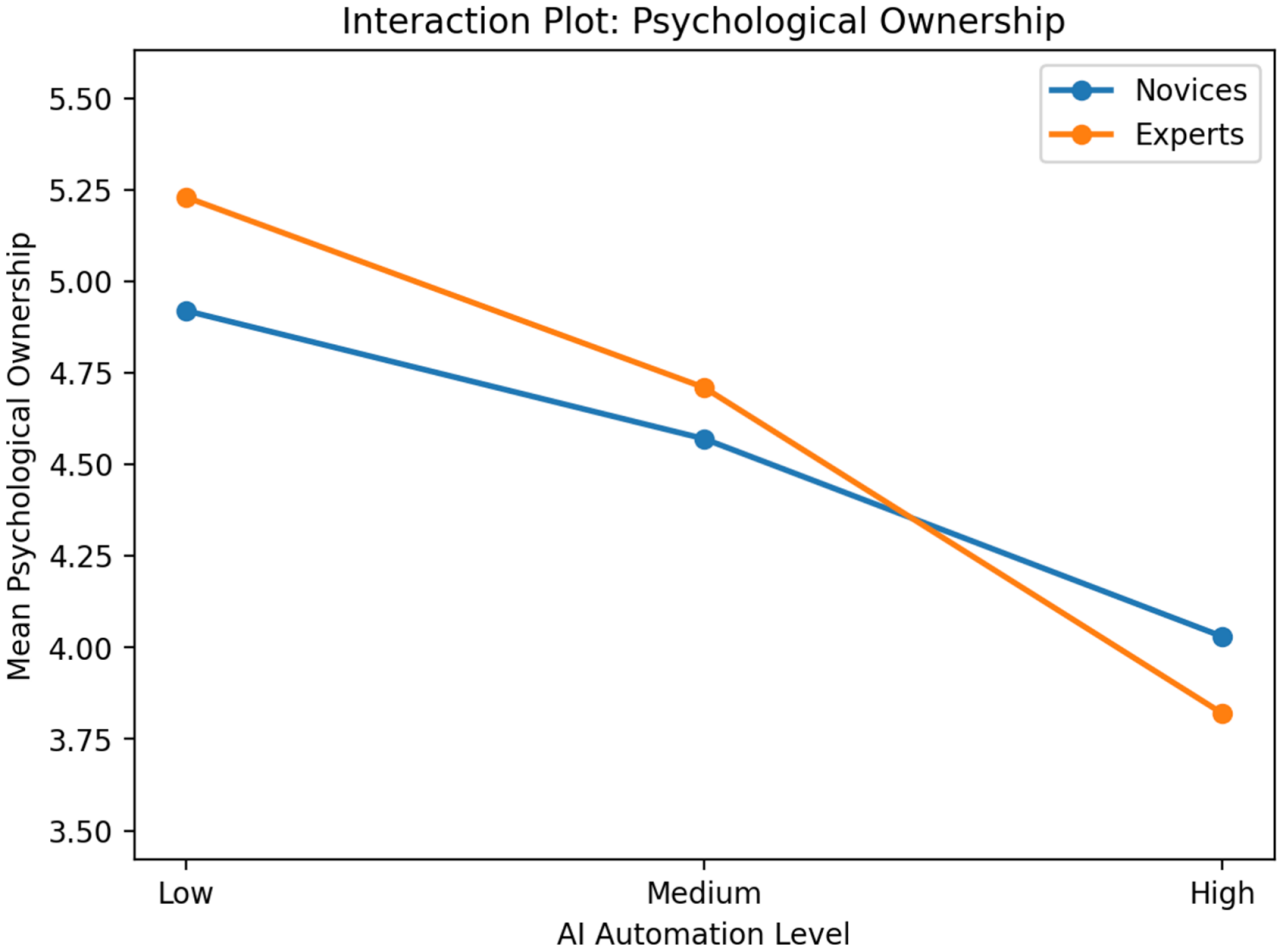
Interaction plot for psychological ownership across AI automation levels and musical expertise groups.

**Figure 4:**
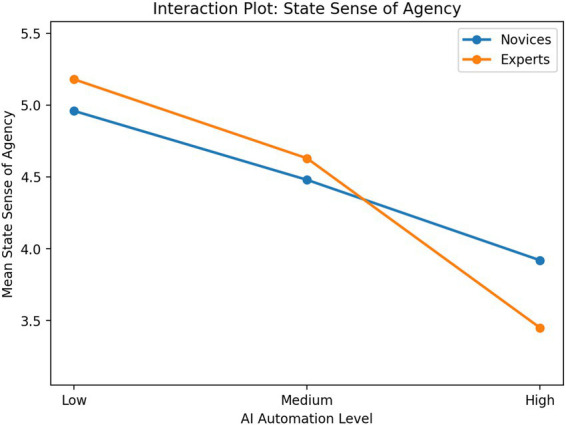
Interaction plot for state sense of agency across AI automation levels and musical expertise groups.

Overall, these findings suggest that as AI autonomy increased, creators’ subjective task load, psychological ownership, and state sense of agency all declined significantly, with this pattern being especially pronounced among experts. These interaction patterns are visualized in Figures 2–4. Across all three outcomes, experts showed a steeper decline than novices as AI automation increased, particularly under the high-automation condition. These results provide initial support for H1 and establish a basis for the subsequent mediation and moderated mediation analyses.

### 4.2 Testing for serial mediation and moderation effects

We used conditional process analysis to test the serial mediating effect of subjective task load and psychological ownership after controlling for DAW familiarity, AI music tool experience, target style familiarity, and general technology acceptance. We also tested the moderating effect of musical expertise level on the first half of the path. We set the number of Bootstrap resamples to 5,000.

Table 3 presents the results of each regression model. In the model with subjective task load as the dependent variable, AI automation level had a significant negative predictive effect. The main effect of musical expertise level was not significant. However, the interaction between AI automation level and musical expertise level was significant. This indicates that the effect of automation level on subjective task load varies depending on musical expertise. Among the control variables, only DAW familiarity reached significance. The model with psychological ownership as the dependent variable showed that AI automation level significantly and negatively predicted psychological ownership. Subjective task load significantly and positively predicted psychological ownership. The interaction between AI automation level and musical expertise level was also significant. This suggests that high automation weakens psychological ownership more obviously in the expert group. The model with state sense of agency as the dependent variable further showed that AI automation level still had a significant negative predictive effect. Both subjective task load and psychological ownership significantly and positively predicted state sense of agency. Psychological ownership had the strongest predictive effect.

**Table 3:** Parameter estimates for the conditional process models.

Predictor	Subjective task load B (SE)	*t*	*p*	Psychological ownership B (SE)	*t*	*p*	State sense of agency B (SE)	*t*	*p*
AI autonomy level	−0.47 (0.09)	−5.22	< 0.001	−0.28 (0.08)	−3.42	0.001	−0.19 (0.07)	−2.79	0.006
Musical expertise level	0.04 (0.11)	0.36	0.721	0.06 (0.10)	0.60	0.548	0.03 (0.09)	0.33	0.744
AI autonomy level × Musical expertise level	−0.31 (0.12)	−2.58	0.011	−0.22 (0.09)	−2.38	0.019	—	—	—
Subjective task load	—	—	—	0.37 (0.07)	5.18	< 0.001	0.14 (0.06)	2.34	0.021
Psychological ownership	—	—	—	—	—	—	0.49 (0.07)	7.16	< 0.001
Familiarity with DAW	0.15 (0.07)	2.18	0.031	0.08 (0.06)	1.31	0.192	0.04 (0.05)	0.78	0.438
Experience with AI music tools	0.07 (0.06)	1.12	0.264	0.05 (0.06)	0.83	0.409	0.06 (0.05)	1.09	0.278
Familiarity with target style	0.09 (0.06)	1.47	0.143	0.07 (0.05)	1.29	0.199	0.05 (0.05)	0.97	0.334
General technology acceptance	0.10 (0.06)	1.61	0.109	0.12 (0.06)	2.01	0.047	0.07 (0.05)	1.34	0.182

Model 1 (subjective task load): *R*^2^ = 0.24, adjusted *R*^2^ = 0.20, *F*(7, 154) = 6.86, *p* < 0.001; Model 2 (psychological ownership): *R*^2^ = 0.43, adjusted *R*^2^ = 0.40, *F*(8, 153) = 14.55, *p* < 0.001; Model 3 (state sense of agency): *R*^2^ = 0.54, adjusted *R*^2^ = 0.52, *F*(8, 153) = 22.44, *p* < 0.001.

Table 4 shows the conditional indirect effect results. For the novice group, the AI automation level significantly affected state sense of agency through the simple mediation path of psychological ownership. It also significantly affected state sense of agency through the serial mediation path of subjective task load to psychological ownership. For the expert group, these two indirect paths were also significant. The effect sizes were larger than those of the novice group. This indicates that high automation has a more obvious negative impact on the expert group. Further testing of the moderated mediation effect revealed that the index of moderated mediation for the serial mediation path was −0.07, with a Bootstrap standard error of 0.03 and a 95% confidence interval of [−0.13, −0.02]. The index of moderated mediation for the simple mediation path of psychological ownership was −0.09, with a Bootstrap standard error of 0.04 and a 95% confidence interval of [−0.18, −0.02]. These confidence intervals did not include 0. This indicates that musical expertise level significantly moderates the indirect effects. Specifically, as the AI automation level increased, the expert group experienced a more obvious decline in subjective task load and psychological ownership. This led to a greater loss of state sense of agency compared to the novice group.

**Table 4:** Conditional indirect effects of AI autonomy level on state sense of agency across levels of musical expertise.

Group	Indirect path	Effect	Boot SE	95% CI
Novice group	AI autonomy level → Psychological ownership → State sense of agency	−0.14	0.05	[−0.26, −0.06]
Novice group	AI autonomy level → Subjective task load → Psychological ownership → State sense of agency	−0.08	0.03	[−0.15, −0.03]
Novice group	Total indirect effect	−0.24	0.07	[−0.38, −0.14]
Expert group	AI autonomy level → Psychological ownership → State sense of agency	−0.23	0.06	[−0.37, −0.12]
Expert group	AI autonomy level → Subjective task load → Psychological ownership → State sense of agency	−0.15	0.04	[−0.25, −0.08]
Expert group	Total indirect effect	−0.38	0.08	[−0.56, −0.25]

Bootstrap resampling = 5,000. Indirect effects are significant when the 95% confidence interval does not include zero.

Overall, the serial mediation and moderation effect test results support H2, H3, and H4. The AI automation level directly reduces creators’ state sense of agency. It also produces significant indirect effects by weakening subjective task load and psychological ownership. Furthermore, this effect is more prominent in individuals with high musical expertise.

## 5 Discussion

This study demonstrates that increasing AI automation levels significantly reduce creators’ subjective task load, psychological ownership, and state sense of agency, particularly among experts. High automation shifts creators from constructors to selectors, weakening their causal attribution and ownership of the outcome (Rafner et al., 2025; Xu et al., 2024; Singh et al., 2025). This aligns with findings that AI involvement alters participation depth and subjective experiences (Choi et al., 2025; Kim et al., 2025). The attenuation of agency results from diminished perceived control and the work no longer feeling like a personal achievement (Xu et al., 2024).

The serial mediation of subjective task load and psychological ownership highlights that excessively low mental demand signifies insufficient investment in key generative stages, further weakening ownership. While AI scaffolding benefits novices, high automation induces deskilling and a professional identity crisis in experts by preemptively handling their stylistic schemas and compositional judgments (Crowston and Bolici, 2024; Wang et al., 2025). Theoretically, this study advances the agency issue in generative AI to a testable mechanistic model, responding to calls for jointly examining control, ownership, and autonomy (Kadenhe et al., 2025; Singh et al., 2025). The present findings also align with recent work suggesting that usability alone is insufficient for preserving authorship-related experience in music AI. In their evaluation of MMM-C, Bougueng Tchemeube et al. (2023) reported generally positive usability and acceptance, while users still raised concerns about controllability and predictability. Similarly, Krol et al. (2025a) show that practising musicians care not only about output quality but also about how co-creative AI fits existing creative practice, whereas Krol et al. (2025b) argue that ownership is better supported when systems depend on the musician’s own input and skill rather than replacing them. Our results extend this literature by quantifying one possible psychological pathway through which such design tensions may matter: as automation increases, reduced task investment may undermine psychological ownership, which in turn diminishes state sense of agency.

Practically, these findings advocate for “agency-preserving AI.” Systems should retain key micro-manipulation permissions and enhance transparency to foster user ownership (Xu et al., 2024; Singh et al., 2025). The present findings should be interpreted in light of several methodological limitations. First, the study relied on a brief, single-session laboratory task involving a 20-min composition of a 30-s segment within one fixed platform configuration. Although this design improved experimental control, it limits ecological validity because real-world music creation is typically iterative, stylistically diverse, and distributed across longer time scales. Second, although AI automation level was experimentally manipulated, the proposed mediators and outcome were all assessed through self-report immediately after task completion. The serial mediation results should therefore be interpreted as evidence of a statistically consistent pathway rather than definitive proof of the real-time psychological process through which agency is altered. Third, the sample consisted of university students, and musical expertise was operationalized through years of formal training; this sharpened group contrast but may not capture the full heterogeneity of contemporary music creators. Fourth, the focal constructs were measured using task-adapted state scales with good internal consistency, but stronger construct-validation procedures and convergent evidence from behavioral indicators—such as edit logs, interaction traces, revision counts, or think-aloud data—would strengthen confidence in the proposed mechanism. Finally, the study examined one task theme and one family of AI tools, so the present pattern should not yet be generalized to other genres, workflows, or co-creative interfaces. Future research should therefore combine longitudinal, higher-ecological-validity designs with multimethod measures of ownership and agency, while also examining design variables such as system transparency and AI personification.

## 6 Conclusion

Although generative AI reduces creative load, it decreases creators’ state sense of agency by weakening psychological ownership. Future music AI design must establish a balance between efficiency improvement and subjectivity preservation.

## Data Availability

The raw data supporting the conclusions of this article will be made available by the authors, without undue reservation.
