# Supplementary material for: Subjective task load and psychological ownership in generative AI collaborative music creation: mechanisms shaping creators’ state sense of agency
Source: Front Psychol. 2026 May 1;17:1835406. doi: 10.3389/fpsyg.2026.1835406 (PMC13177856; doi:10.3389/fpsyg.2026.1835406)
Supplement: Supplementary file 1 [file Supplementary_file_1.docx]

**Appendix A. Adapted Task-Contextualized Measurement Scales**

All focal measures were rated on a 7-point Likert scale (1 = strongly disagree, 7 = strongly agree). Higher scores indicated higher levels of the focal construct unless otherwise noted. Reverse-scored items are marked with (R).

**A1. Subjective Task Load**

Adapted from the Mental Demand and Effort dimensions of the NASA-TLX for the present music co-creation task.

1. This task required substantial mental effort from me.
2. I had to concentrate intensely to complete this task.
3. I found the music creation process cognitively demanding.
4. I had to work hard to keep the composition process moving.
5. Completing this task required sustained effort.
6. I felt mentally taxed while finishing this piece.

**A2. Psychological Ownership**

Adapted as a state-based measure of felt ownership over the music created in the task.

1. I felt that the final piece was my creation.
2. I felt a strong sense that this work belonged to me.
3. This music piece felt personally mine.
4. I felt attached to the piece I created.
5. I felt that my ideas were reflected in the final outcome.
6. I felt a sense of ownership over the final piece.

**A3. State Sense of Agency**

Adapted from the state-based measurement logic of the SOARS for the present music co-creation task.

1. I felt that I was in control of the music creation process.
2. I felt that my actions directly shaped the final piece.
3. I felt that the final outcome followed from my own decisions.
4. I felt that I was the author of what was created.
5. I felt able to intentionally influence how the piece developed.
6. I felt that the resulting piece reflected what I meant to create.
7. The final piece seemed to happen by itself rather than because of me. (R)
8. I felt more like an observer than an agent during the creation process. (R)
